# Prevalence of colistin resistance gene *(mcr-1)* containing *Enterobacteriaceae* in feces of patients attending a tertiary care hospital and detection of a *mcr-1* containing, colistin susceptible *E*. *coli*

**DOI:** 10.1371/journal.pone.0178598

**Published:** 2017-06-02

**Authors:** Elisabeth M. Terveer, Roel H. T. Nijhuis, Monique J. T. Crobach, Cornelis W. Knetsch, Karin E. Veldkamp, Jairo Gooskens, Ed J. Kuijper, Eric C. J. Claas

**Affiliations:** Department of Medical Microbiology, Leiden University Medical Center, Leiden, the Netherlands; Institut National de la Recherche Agronomique, FRANCE

## Abstract

The emergence of the plasmid-mediated *mcr* colistin resistance gene in the community poses a potential threat for treatment of patients, especially when hospitalized. The aim of this study was to determine the prevalence of all currently known *mcr* mediated colistin resistance gene in fecal samples of patients attending a tertiary care hospital. From November 2014 until July 2015, fecal samples of patients attending the Leiden University Medical Center were collected and screened for presence of *mcr* using real-time PCR. Two of 576 patients were positive for *mcr-1*, resulting in a prevalence of 0.35%, whereas no *mcr-2* was found. One of these samples was culture negative, the second sample contained a *bla*CMY-2 and *mcr-1* containing *E*.*coli*. This strain belonged to Sequence Type 359 and serotype O177:H21. The *mcr-1* containing *E*.*coli* was phenotypically susceptible to colistin with a MIC of ≤ 0.25mg/l, due to a 1329bp transposon IS10R inserted into the *mcr-1* gene as identified by WGS. This prevalence study shows that *mcr-1* is present in low levels patients out of the community attending a hospital. Furthermore the study underlines the importance of phenotypical confirmation of molecular detection of a *mcr-1* gene.

## Introduction

Colistin, also known as polymyxin E, is highly active against most Gram-negative bacteria [1]. However, its nephrotoxicity and neurotoxicity has prevented the use of colistin in regular patient treatment [2]. Therefore, colistin usage was mainly limited to veterinary medicine for treatment of gastrointestinal infections in food producing animals [3]. In the Netherlands, colistin is frequently used for selective gut decontamination in Intensive Care Unit (ICU) and stem cell transplantation patients [4, 5]. Colistin regained new worldwide interest after the emergence of multi-drug resistant (MDR) *Enterobacteriaceae* and is nowadays used as a last resort antibiotic for infections caused by MDR *Enterobacteriaceae*. The recent finding of a plasmid harbouring a novel colistin resistance gene, *mcr-1* and *mcr-2*, is therefore of concern [6, 7].

The *mcr-1* colistin resistance gene is predominantly found in *Enterobacteriaceae*, and results in a moderate level of resistance, with MIC values varying from 4 to 16 mg/l [8, 9]. The prevalence of *mcr-1* varies considerably and ranges from 0.02% to 20.6% in livestock, 1.3% to 19% in retail meat and 0.08% to 2% in hospitalized patients [10–14]. The worldwide distribution of the *mcr-1* gene and a relatively high prevalence of *mcr-1* mediated colistin resistance in livestock and retail meat suggests food animals as reservoir for transmission to humans [8]. Until now, almost exclusively Extended Spectrum Beta-Lactamase (ESBL) producing or colistin resistant isolates have been screened for the presence of *mcr-1*. A number of reports of *mcr-1* in the United States of clinical and ESBL-negative strains indicate that the true extent of *mcr-1* prevalence amongst unselected Gram-negatives may be highly underestimated [15, 16]. The *mcr-2* colistin resistance gene had 76.7% nucleotide identity to *mcr-1* and had so far only been found in colistin-resistant *E*.*coli* isolates identified from porcine and bovine [7].

Studies about human fecal carriership of *mcr-1* in the community are limited, and so far only been described in China; in healthy volunteers (prevalence of 19 of 2923 = 0.65%), in a public bacterial metagenome dataset before 2010 (prevalence of 3/1267 = 0.24%) [17, 18], and in Dutch travellers returning from Asia, South America or Africa (prevalence of 0.95%–4.9%) [19, 20]. Recently, no *mcr* genes could be detected in the stool of 1091 healthy Swiss individuals [21]. Epidemiological data on the prevalence of *mcr-*1 in the community attending a hospital are lacking and the risk of colonized patients to spread *mcr-1* positive bacteria is unknown. Therefore, the aim of this study was to determine the prevalence of *mcr* mediated colistin resistance gene in fecal samples of patients attending a tertiary care hospital.

## Material/methods

### Patients and specimens

Between November 2014 and July 2015, fecal samples were obtained from patients on admittance to internal medicine and surgical wards, and from patients attending the kidney transplant outpatient clinic of the Leiden University Medical Center (LUMC) in the Netherlands. These wards were selected for their relative high patient turn-over, enabling more rapid inclusion of sufficient patients attending our hospital. The fecal samples were originally used for a study to define the role of *Clostridium difficile* in asymptomatic colonised patients at admittance to the hospital. The samples were processed within 72 hours of arrival at the laboratory and were subsequently stored at -20°C, without addition of glycerol. These samples obtained for *C*. *difficile* screening were also used for screening of the *mcr* gene. The medical ethical committee “Medisch Ethische Toetsings Commissie” of the LUMC waived the need for consent for the additional analysis on these fecal samples.

### DNA extraction and real-time PCR

After thawing the stored fecal samples, DNA extraction was performed using the MagnaPure96 system (Roche Diagnostics, Almere, Netherlands). In short, approximately 0.3 to 0.4 gram (half a pea) feces was resuspended in 1mL S.T.A.R. buffer (Roche Diagnostics, Almere, The Netherlands), supplemented with Precellys beads (Bertin Technology, France), mixed thoroughly by shaking on a Vibrax shaker (5 min, 2200rpm) and centrifuged for 1min at 14000 rpm. Of the supernatant, 200μl was used for nucleic acid (NA) extraction using the MP96 system and Viral NA Small volume kit (Roche Diagnostics) yielding a final eluate of 100μl. To monitor the NA extraction process and the presence of potential PCR inhibitors in the eluate, an universal internal control Phocine Herpes Virus (PhHV) was used [22]. Initially real-time PCR for the specific detection of the *mcr-*1 gene was tested in a multiplex assay with PhHV as described previously [23]. After the report of Xavier *et al*., describing *mcr-2*, a generic *mcr* real-time PCR assay for the detection of both *mcr-1* and *mcr-2* was developed and used to screen for the presence of additional *mcr-2* containing samples (Table 1) [7].

**Table 1:** Primers and probe used to screen for the presence of *mcr*-genes.

Oligonucleotide	Sequence (5’-3’)	PCR product
*Mcr*-generic fw	GCCAAATACCAAGAAAATG	98bp
*Mcr-*generic probe	TATCACGCCACAAGATAC	
*Mcr-*generic rev	TTATCCATCACGCCTTTT	

### Culture and colistin susceptibility testing

To further characterize *mcr* containing isolates, *mcr* positive fecal samples were cultured on commercially available sheep blood-, CNA- (colistin and naladixic acid containing agar) and CLED- (cysteine lactose electrolyte deficient) medium (BioMérieux, Marcy l’Etiole, France) both directly and after enrichment in a Tryptic Soy Broth with and without colistin (2 mg/l). All morphological different aerobic Gram-negative bacteria were identified by MALDI-TOF MS (Microflex, Bruker Daltonics, Bremen, Germany) and tested for the presence of *mcr-1* by real-time PCR as described earlier. All bacterial isolates were also tested for colistin resistance with VITEK2 (card N199, BioMérieux, Marcy l’Etiole, France) and Sensititre colistin microdilution assay (Sensititre, TREK Diagnostic Systems, Inc., Cleveland, OK), using EUCAST breakpoints for *Enterobacteriaceae*, which interprets a MIC of ≤ 2 mg/l as susceptible and > 2mg/l as resistant.

### Whole Genome Sequence analysis

Whole Genome Sequence analysis of *mcr-1* containing isolates was performed to further characterize the *E*. *coli* strain including the plasmid carrying the *mcr-1* gene and other genes associated with antimicrobial resistance [6]. The genome sequence of the *mcr-1* containing isolate was determined using the Pacific Biosciences *RS*II system from DNA prepared by the Qiagen Genomic Tip 500/G kit (Qiagen, Hilden, Germany) following the manufacturer’s recommendations. *De novo* assembly was performed using SMRT®Analysis v2.3.0 (PacBio's bioinformatics software suite) with expected genome size of 5 Mbp and coverage of 30. The assembled sequence was analysed using Geneious software V8.0.5 (Biomatters, Auckland, New Zealand) and the online tools Resfinder, MLST, SeroTypeFinder and Plasmidfinder (http://genomicepidemiology.org/). The plasmid sequence was analysed in DNA plotter to generate a circular DNA map.

## Results

### *Mcr* prevalence and culture of *mcr* containing isolates

A total of 621 fecal samples of 576 unique patients were screened for presence of the *mcr* genes by real-time PCR. The median age of patients at submission of their stool was 62 years (range 18–93). Two samples of two different patients (0.35%) were positive for *mcr-1* in real-time PCR with quantification cycle (Cq) values of 31 and 17, respectively (S1 Table). Additional testing with the *mcr*-generic real-time PCR assay confirmed this finding and did not find extra positive samples. A *mcr-1* containing *E*.*coli* isolate was cultured from the second fecal sample (Cq 17) only, in subcultures of the enrichment broth without colistin. Remarkably, despite the presence of *mcr-1* gene sequences, this *E*.*coli* isolate tested colistin susceptible (MIC <0.25 mg/l), which was confirmed in triplicate by both VITEK2 and the Sensititre assay. The antimicrobial susceptibility results and the corresponding genes coding for resistance are depicted in Table 2. Because of the decreased susceptibility to cephalosporins, the production of ESBL was tested phenotypically using the combination disk diffusion test, with a negative result. Subsequent testing for an AmpC β-lactamase gene by an in-house developed real-time PCR assay showed the presence of the *bla*CIT gene.

**Table 2:** Antibiotic phenotype with the corresponding molecular resistance of cultured *mcr-1* containing *E*.*coli*.

Antibiotic	MIC (mg/l)	Interpretation	Encoding resistance genes
Ampicillin	≥ 32	R	blaTEM-1B
Amoxicillin/ Clavulanic acid	≥ 32	R	blaTEM-1B
Cefuroxime	32	R	blaCMY-2
Cefotaxime	4	R	blaCMY-2
Cefoxitin	≥ 32	R	blaCMY-2
Ceftazidime	16	R	blaCMY-2
Cefepime	≤ 1	S	
Ciprofloxacin	≥ 4	R	
Colistin	≤ 0.25	S	*Mcr-1* inserted by IS10R transposon
Gentamicin	≤ 1	S	*aph(3’)-lc*, *strB*, *strA*, *aadA5*
Meropenem	≤ 0.25	S	
Nitrofurantoin	≥ 320	S	
Piperacillin/Tazobactam	≤ 4	S	
Tetracycline	128	R	*tetB*
Tobramycin	≤ 1	S	*aph(3’)-lc*, *strB*, *strA*, *aadA5*
Trimethoprim/ Sulfamethoxazole	≥ 4	R	*sul1*, *sul2*, *dfraA17*

The phenotype was tested with VITEK2 and a colistin microdilution assay, using EUCAST breakpoints. Molecular resistance determined with whole genome sequencing.

### Whole Genome Sequence analysis

**Fig 1:**
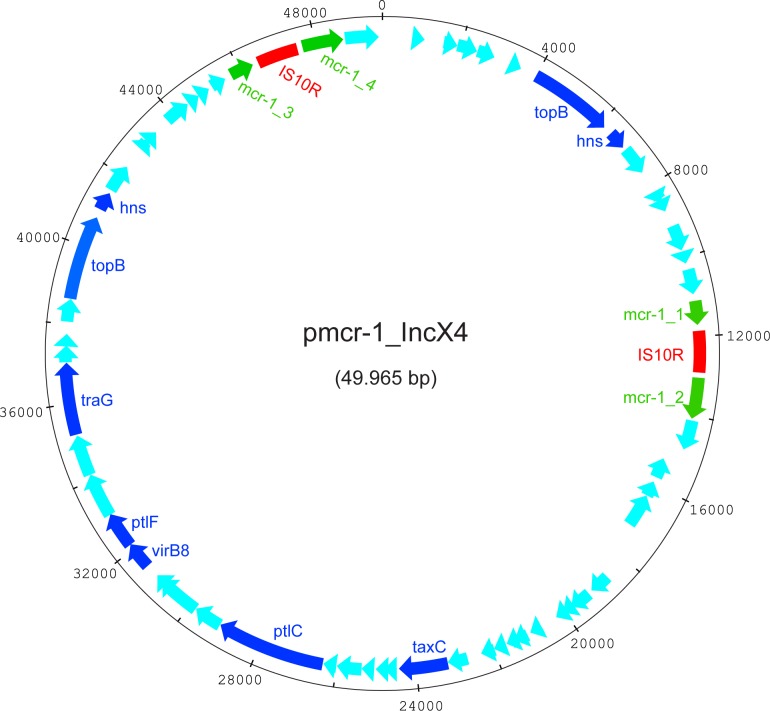
Circular presentation of the *mcr-1* containing *IncX4* plasmid in the colistin susceptible *E*.*coli*. In green the *mcr-1* sequence. In red the IS10R insertion sequence, interrupting the *mcr-1* gene at position 572. Arrows indicate open reading frames (ORFs), dark blue ORFs with annotation, light blue ORFs without annotation (hypothetical protein). Numbers indicate nucleotide positions.

WGS analysis showed that the *mcr-1* gene found in the colistin susceptible *E*. *coli* isolate had a homology of 100% with the first published *mcr-1* gene sequence [6]. However, the reading frame was disrupted by a 1329bp long IS10R transposon (Fig 1). WGS analysis of the *E*.*coli* resulted into six contigs with a total length of ~5.5 Mbp (accession numbers: CP016546-CP016551, S2 Table). The largest contig was ~5.1 Mbp, covering the expected *E*. *coli* genome size, whereas analysis of the remaining five contigs (length between ~7.3 kb and ~126 kb) with PlasmidFinder 1.3 indicated the presence of plasmids IncX4 (~50kb), IncI2 (~86kb), IncB/O/K/Z (~91kb) and IncY (~126kb). WGS analysis also revealed the presence of two identical IS10R containing *mcr-1* genes located on the same IncX4 plasmid. Multi Locus Sequence Typing (MLST) and serotype analysis showed that the *E*.*coli* belonged to Sequence Type (ST) 359 and serotype O177:H21. With ResFinder, the AmpC belonging to the CIT-group, as detected by the in-house AmpC real-time PCR, was confirmed to be present as *blaCMY-2*, located on the plasmid designated as IncB/O/K/Z. Additional genes associated with antimicrobial resistance detected in the sequence with their resulting antimicrobial phenotype are depicted in Table 2.

### Patient characteristics

Both *mcr-1* positive patients were kidney transplant patients. The *mcr-1* positive stool sample from which no *mcr-1* containing isolate could be cultured belonged to a patient admitted to the acute care ward due to bacteremia with a colistin resistant *Salmonella enterica* serotype *Dublin* (MIC ≥4 mg/l). The *S*. *enterica* isolate tested negative with the specific *mcr-1* PCR. The feces with the colistin susceptible *mcr-1 E*.*coli* belonged to a patient attending the kidney transplant outpatient clinic. No epidemiologic link could be established between the two patients. The patients did not have a history of recent travelling and had not been treated with colistin recently. Also, none of the patients had developed an infection with a *mcr-1* containing isolate.

## Discussion

To assess the risk of *mcr* introduction into our academic tertiary care hospital, the prevalence of *mcr* in fecal samples obtained from patients attending our hospital was investigated and found to be 0.35% (n = 2) of the 576 tested patients for *mcr-1*, whereas no *mcr-2* was found. This low prevalence is in accordance with earlier studies performed in asymptomatic carriers in the European community, ranging from 0% to 0.92% [19–21]. However, studies on *mcr-1* prevalence in asymptomatic carriers attending a hospital are lacking. Infections in hospitalized patients with *mcr-1* positive isolates have been reported in a number of countries, ranging from 0.24% to 1.4% depending on the used denominator [6, 10, 24]. In line with earlier studies, no *mcr-2* containing samples were detected in this study [21, 25].

One of the *mcr-1* positive fecal samples from the current study could not be confirmed by culture, most likely due to the fecal storage without glycerol at -20°C for one year which reduces the viability of Gram-negative bacteria. The fecal sample of the second patient contained a *mcr-1* positive *E*.*coli* with a colistin MIC of <0.25 mg/l. WGS analysis of the isolate revealed the presence of IS10R, encoding for an active transposon commonly found in *Enterobacteriaceae* [26]. Introduction of this IS10R into the *mcr-1* gene resulted in a non-functional *mcr-1* gene. Interestingly, two identical *mcr-1* genes with IS10R duplicates were located on *IncX4*, a plasmid that has been frequently observed in combination with *mcr-1* [10, 12, 27, 28]. The *mcr-1* containing *E*.*coli* belonged to ST359, this ST with a very similar antimicrobial resistance pattern is earlier described on chicken retail meat in Denmark [10]. Though we tested all morphological different Gram negative *Enterobacteriaceae* for the presence of *mcr-1*, we cannot exclude the possibly that more than one *mcr-1* containing bacterial species was present in the positive tested feces samples.

Pham Thanh *et al*. reported the first *mcr-1* positive but colistin susceptible isolate, a *Shigella sonnei*, that was based on a truncated *mcr-1* gene caused by a 22bp duplication [29]. A colistin susceptible *mcr-1* containing *E*.*coli* isolate with unknown cause of the susceptibility was reported in August 2016 by Liassine *et al*. [25]. Although the altered *mcr-1* gene of the *Shigella sonnei* could be re-activated by conjugation experiments resulting in a colistin resistant phenotype, the *mcr-1* gene interrupted with IS10R containing *E*.*coli* of this study cannot be re-activated, as upon removal of an IS transposon two remaining nucleotides would disrupt the reading frame of the gene [30]. These studies underline the importance of phenotypical confirmation after molecular screening, as respectively the *E*.*coli* and *Shigella sonnei* isolate showed colistin susceptibility despite the presence of *mcr-1* gene sequences that had been detected by PCR amplification.

The *mcr-1* positive *E*.*coli* isolate showed resistance to third generation cephalosporins due to the presence of a AmpC β-lactamase gene, *blaCMY-2*, as has previously been found by Prim *et al*. [24]. As almost all earlier studies only screened for the presence of *mcr-1* in ESBL producing isolates, the true extent of the *mcr-1* prevalence may be underestimated [10, 11, 14, 19, 31, 32].

Most likely, the kidney transplant patients acquired the *mcr-1* gene in the community, for instance by consumption of *mcr-1* containing retail meat [6, 10, 12, 13, 32]. Spread of the *mcr-1* gene in the community and successively in the hospital would pose a threat to patients developing an infection with *mcr-1* containing multidrug resistant isolates. *Enterobacteriaceae* resistant to both carbapenems and colistin by the presence of plasmid mediated *mcr-1* have already been reported [17, 31, 33–35]. Therefore, screening for and isolation of *mcr-1* containing patients should be considered. Prudence and close monitoring is necessary, especially when selective gut decontamination with colistin for ICU and hematological stem cell patients is common practice.

In conclusion, the current prevalence of *mcr-1* suggests that spread from the community into the hospital environment is low, but cannot be excluded. Furthermore the finding of a colistin susceptible, *mcr-1* containing *E*.*coli* underlines the importance of phenotypical confirmation after molecular screening.

## Supporting information

S1 Table *Mcr* real-time PCR and culture results of all 621 screened fecal samples. (XLSX)

S2 Table Accession numbers of the six contig containing *mcr-1* positive, colistin susceptible *E*.*coli*. (XLSX)
